# MR image mimicking the “eye of the tiger” sign in Wilson’s disease

**DOI:** 10.1007/s00415-014-7322-y

**Published:** 2014-03-26

**Authors:** T. Litwin, M. Karlinski, M. Skowrońska, K. Dziezyc, M. Gołębiowski, A. Członkowska

**Affiliations:** 1 Second Department of Neurology, Institute of Psychiatry and Neurology, Sobieskiego 9, 02-957 Warsaw, Poland; 2 Department of Radiology, Medical University, Warsaw, Poland; 3 Department of Experimental and Clinical Pharmacology, Medical University, Warsaw, Poland

Dear Sirs,

The eye of the tiger sign (EOT), seen on T2 sequences of magnetic resonance imaging (MRI), is considered pathognomonic for neurodegeneration with brain iron accumulation type 1 (NBIA1, previously known as Hallervorden–Spatz syndrome) [1–3]. This sign is described as low signal intensity in both globi pallidi (due to iron accumulation) that surrounds a central region of high signal intensity (caused by gliosis, edema, and neuronal loss with subsequent secondary vacuolization and cavitation) [1–3]. It has been occasionally reported in neurodegenerative disorders other than NBIA1, including neuroferritinopathy, progressive supranuclear palsy, multisystem atrophy, pure akinesia, and corticobasal degeneration [1–3]. Here, we describe the first case of EOT-like MRI appearance in a 41-year-old man with Wilson’s disease.

At the age of 24 years, the patient developed dysarthria, oromandibular dystonia, gait disturbances, and positional hand tremor. One year later he was diagnosed with Wilson’s disease, based on the presence of Kayser–Fleischer ring and abnormal copper metabolism [i.e. decreased serum ceruloplasmin (8.2 mg/dL, normal 25–45) and serum copper (30 μg/dL, normal 70–140 μg/dL) combined with increased copper urinary excretion (664 μg/24 h, normal 0–50)]. Genetic examination confirmed compound heterozygosity, with *ATP7B* mutations in exon 13 (G988R) and in exon 7 (IVS7 + 3A>G). After introduction of 1000 mg/day d-penicillamine, the patient gradually improved. In 2013 his remaining symptoms were only slight dysarthria and oromandibular dystonia (occasionally grimacing). The Kayser–Fleischer rings were no longer present. However, a routine control brain MRI revealed abnormalities very similar to EOT sign (Fig. 1).

**Fig. 1:**
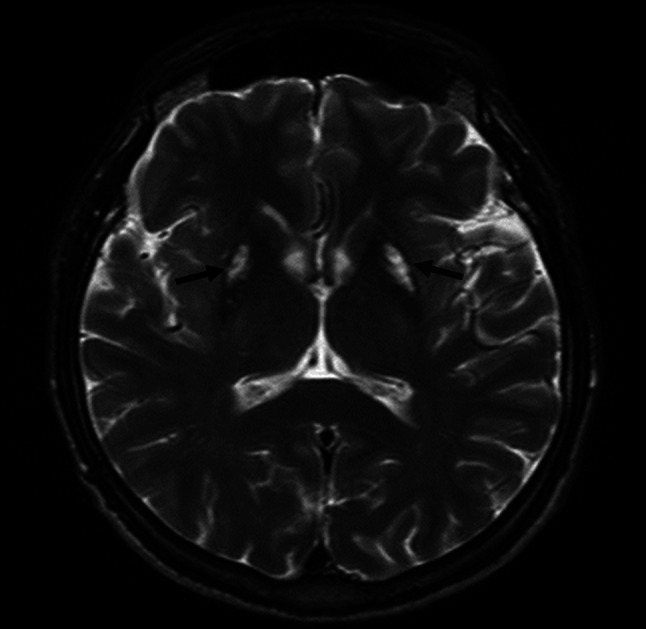
The MR image mimicking the “eye of the tiger” sign in T2-weighted magnetic resonance sequences; bilateral hypointensity in both putaminal regions and lateral parts of globi pallidi, with a central hyperintensity (*black arrows*)

The difference was that hypointensity tended to localize in the lateral part of the globus pallidus and putamen instead of the medial part of the globus pallidus. Subsequent MR spectroscopy showed that the mean *N*-acetyloaspartate/creatine ratio (TE-144mS) in the globus pallidus was 1.55, and the mean choline/creatine ratio (TE-144 mS) was 1.10 (Fig. 2a). In the normal appearing control region, the values were 2.37 and 1.08, respectively (Fig. 2b), which may reflect neuronal loss in the affected area. Noteworthy, the patient has no co-morbidities, does not drink alcohol and had no occupational risk factors for tissue metal accumulation. Normal levels of serum iron metabolism markers (ferritin, iron, total iron binding capacity) combined with the early age of the disease onset, lack of anemia and diabetes allowed to exclude aceruloplasminemia and neuroferritinopathy. The lack of acanthocytosis, pigmentary retinopathy, hypoprobetalipoproteinemia, and good response to d-penicillamine with no subsequent progression over 15 years allowed us to exclude NBIA1. The patient's brother was diagnosed with hepatic Wilson’s disease without any neurological signs at 28 years of age.

**Fig. 2:**
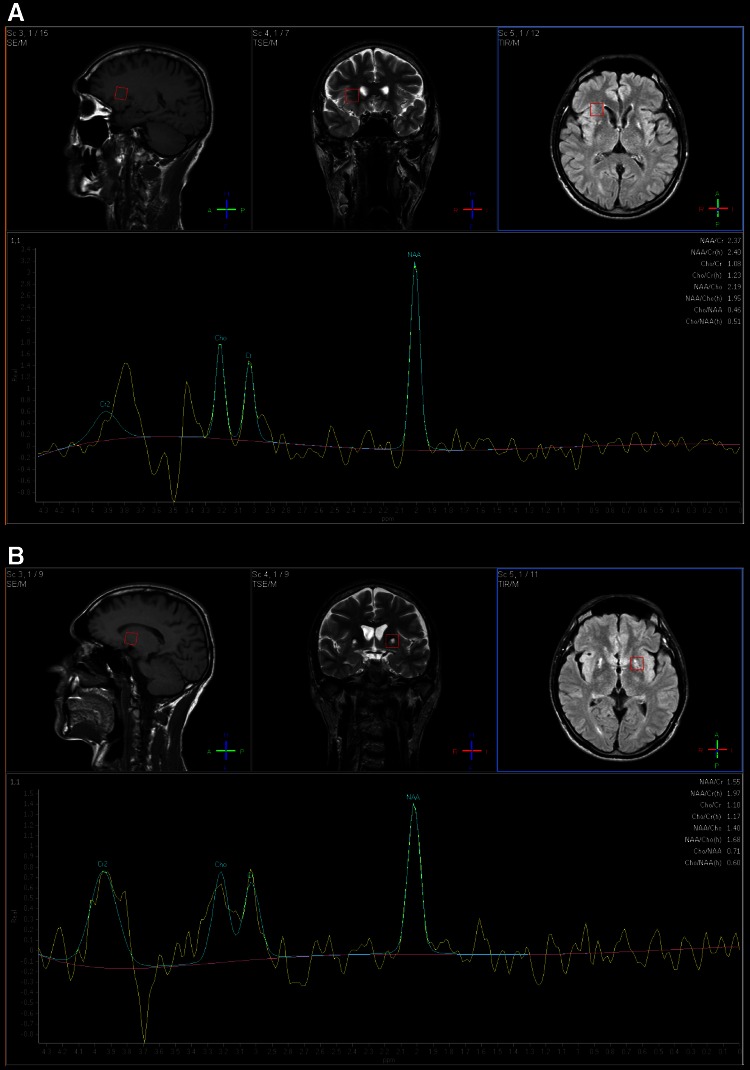
The magnetic brain spectroscopy from: **a** unaffected structures in the right temporal lobe; **b** central, hyperintensive part of “eye of the tiger” sign showing markedly decreased *N*-acetyloaspartate in the globus pallidus as a results of reactive gliosis

Wilson’s disease is a treatable, inherited disorder leading to pathological copper accumulation that damages affected organs, especially the liver and brain. It has been suggested that disturbances of iron metabolism occur in the liver of Wilson’s disease patients, as copper-dependent enzymes are also involved in its metabolic pathways [4]. In a recent post-mortem study we demonstrated that iron accumulates also in the brain [5]. As the EOT sign suggests accumulation of iron [2, 3, 6, 7], its increased concentration in the basal ganglia of the reported patient appeared to be particularly pronounced. However, the exact role of iron-metabolism disturbances in Wilson’s disease is still unknown and further studies are needed. Considering that patients with EOT sign may occasionally have conditions other than NBIA1, it is advisable to carry out careful differential diagnosis in order not to miss a condition that can be effectively treated.

## References

[1] Chang CL, Lin CM (2011). Eye-of-the tiger sign is not pathognomonic of pantothenate kinase-associated neurodegeneration in adult case. Brain Behav.

[2] Dusek P, Jankovic J, Le W (2012). Iron dysregulation in movement disorders. Neurobiol Dis.

[3] Ikeda T, Matsuo Y, Ueda A, Hirano T (2013). Pseudo eye of the tiger sign in atypical Parkinsonism. Neurol Sci.

[4] Hayashi H, Motoyoshi Y, Yoshikazu F, Wakusawa S (2006). Compound overload of copper and iron in patients with Wilson’s disease. Med Mol Morphol.

[5] Litwin T, Gromadzka G, Szpak GM, Jablonka-Salach E, Bulska A, Czlonkowska A (2013). Brain metal accumulation in Wilson’s disease. J Neurol Sci.

[6] Van den Bogaard SJA, Kruit MC, Dumas E, Roos RAC (2014). Eye-of-the-tiger-sign in a 48 year healthy adult. J Neurol Sci.

[7] Kumar N, Boes JC, Babovic-Vuksanovic D, Boeve BF (2006). The “eye-of-the-tiger” sign is not pathognomonic of the PANK2 mutation. Arch Neurol.

